# P-1699. Antimicrobial Administration and 30-Day Outcomes in Patients Hospitalized with MRSA-Related Infections who Self-Discharge

**DOI:** 10.1093/ofid/ofae631.1865

**Published:** 2025-01-29

**Authors:** Julia Lewis, Kevin Nechodom, Makoto M Jones

**Affiliations:** Salt Lake City VA Medical Center, Salt Lake City, Utah; University of Utah Division of Epidemiology, Salt Lake City, Utah; Veterans Affairs, Salt Lake City, Utah

## Abstract

**Background:**

Management of serious infectious diseases typically includes hospitalization and IV antibiotics, particularly where methicillin-resistant *Staphylococcus aureus* (MRSA) is the causative organism. When patients with these infections decide to self-discharge, it presents a challenging clinical decision for their providers. Here we describe antimicrobial prescribing practices for patients hospitalized with different infectious disease diagnoses and their 30-day outcomes.

**Figure:**
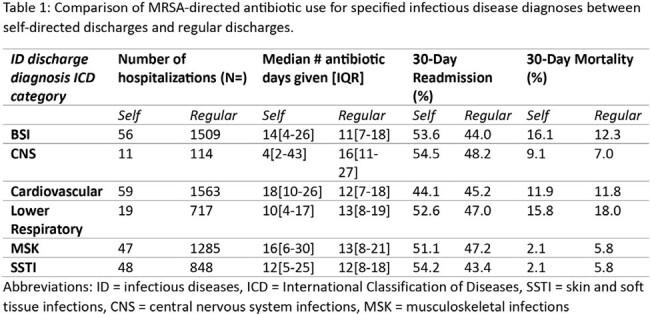

**Methods:**

This retrospective cohort study included patients 18 years and older admitted between January 1, 2010, and December 31, 2019, with admission blood cultures growing MRSA. Veterans Affairs (VA) electronic health data was extracted from the Veterans Informatics and Computing Infrastructure, microbiology data, inpatient antibiotic administration, discharge antibiotic prescriptions, ICD-9- and ICD-10-CM coded infectious disease discharge diagnoses, discharge type (self-discharge or regular discharge), as well as 30-day all-cause mortality and readmission. Descriptive statistics were used to compare antibiotic prescribing for specific infectious diseases between self- and regular discharges.

**Results:**

While hospitalizations with patients self-discharging were lower for each infectious disease, their total antibiotic days were similar for most listed MRSA-related infections. However, for 30-day readmission rates, there was a higher rate among self-discharged patients for most all infections except cardiovascular. Conversely, 30-day mortality appeared to be higher among regular discharges for most infectious disease diagnoses except central nervous system and bloodstream infections.

**Conclusion:**

While this analysis is preliminary, there is a concerning trend towards higher 30-day readmissions for patients with MRSA infections who self-discharge, despite similar durations of MRSA-directed antibiotic prescribing. This suggests additional factor(s) than antibiotic administration that play a role in readmissions for this patient population. Further research is needed to elucidate what factors contribute to readmission for patients who self-discharge from hospitalization for serious MRSA-related infections.

**Disclosures:**

**All Authors**: No reported disclosures

